# Pennsylvania State Veterinary Medical Association

**Published:** 1893-03

**Authors:** 


### EDITORIAL.

### PENNSYLVANIA STATE VETERINARY MEDICAL ASSOCIATION.

The Pennsylvania State Veterinary Medical Association held its annual meeting in Philadelphia on March 7th and 8th. Our readers will find in this number of the JOURNAL several of the valuable papers which were read at this meeting, and in the next number will have a full account of the proceedings for which we could not longer hold the publication of the March issue. This meeting was preëminently one of the best veterinary conventions ever held in the country. It was held in the rooms of the College of Physicians, which accords the veterinarian his proper recognition as a medical confrere.

The sessions were largely attended by a half-hundred members from all over the State, and by visitors from other States. The discussions were entered into by sanitarians and prominent members of the Board of Health and by eminent medical men. The question of milk supply and the relation of the veterinarian to the question of public health formed important portions of the reports and discussions.

The President, Dr. W. H. Hoskins, deserves the greatest credit for the training of the Pennsylvania Association and the great success which it has achieved, and is to be congratulated by the members of the United States Veterinary Medical Association who have already recognized his valuable work in his position as their Secretary.

